# Supplementary material for: Construction and Validation of a Prognostic Gene-Based Model for Overall Survival Prediction in Hepatocellular Carcinoma Using an Integrated Statistical and Bioinformatic Approach
Source: Int J Mol Sci. 2021 Feb 5;22(4):1632. doi: 10.3390/ijms22041632 (PMC7915780; doi:10.3390/ijms22041632)
Supplement: Supplementary file 1 [file ijms-22-01632-s001.pdf]

International Journal of
Molecular Sciences

MDPI

Article

Construction and Validation of a Prognostic Gene-Based Model for Overall Survival Prediction in Hepatocellular Carcinoma Using an Integrated Statistical and Bioinformatic Approach

Eskezeia Y. Dessie [1], Siang-Jyun Tu[2], Hui-Shan Chiang[2], Jeffrey J.P. Tsai[1], Ya-Sian Chang[2,*], Jan-Gowth Chang[2,*] and Ka-Lok Ng [1,3,4,*]

1 Department of Bioinformatics and Medical Engineering, Asia University, Taichung, Taiwan; No. 500, Lioufeng Rd., Wufeng, Taichung 41354, Taiwan; estu2003@gmail.com (E.Y.D.); jjptsai@gmail.com (J.-P.T.); ppiddi@gmail.com (K.-L.N.)
2 Department of Laboratory Medicine, and Center for Precision Medicine, China Medical University and Hospital, Taichung, Taiwan; No. 2, Yude Rd., North Dist., Taichung City 404332, Taiwan; t34752@mail.cmuh.org.tw (S.-J.T.); d18448@mail.cmuh.org.tw (H.-S.C.); t25074@mail.cmuh.org.tw (Y.-S.C.); d6781@mail.cmuh.org.tw (J.-G.C.)
3 Department of Medical Research, China Medical University Hospital, China Medical University, Taichung, Taiwan; No. 2, Yude Rd., North Dist., Taichung City 404332, Taiwan; ppiddi@gmail.com (K.-L.N.)
4 Center for Artificial Intelligence and Precision Medicine Research, Asia University, Taichung, Taiwan; No. 500, Lioufeng Rd., Wufeng, Taichung 41354, Taiwan; ppiddi@gmail.com (K.-L.N.)
* Correspondence: t25074@mail.cmuh.org.tw (Y.-S.C.); ppiddi@gmail.com (K.-L.N.); d6781@mail.cmuh.org.tw (J.-G.C.)

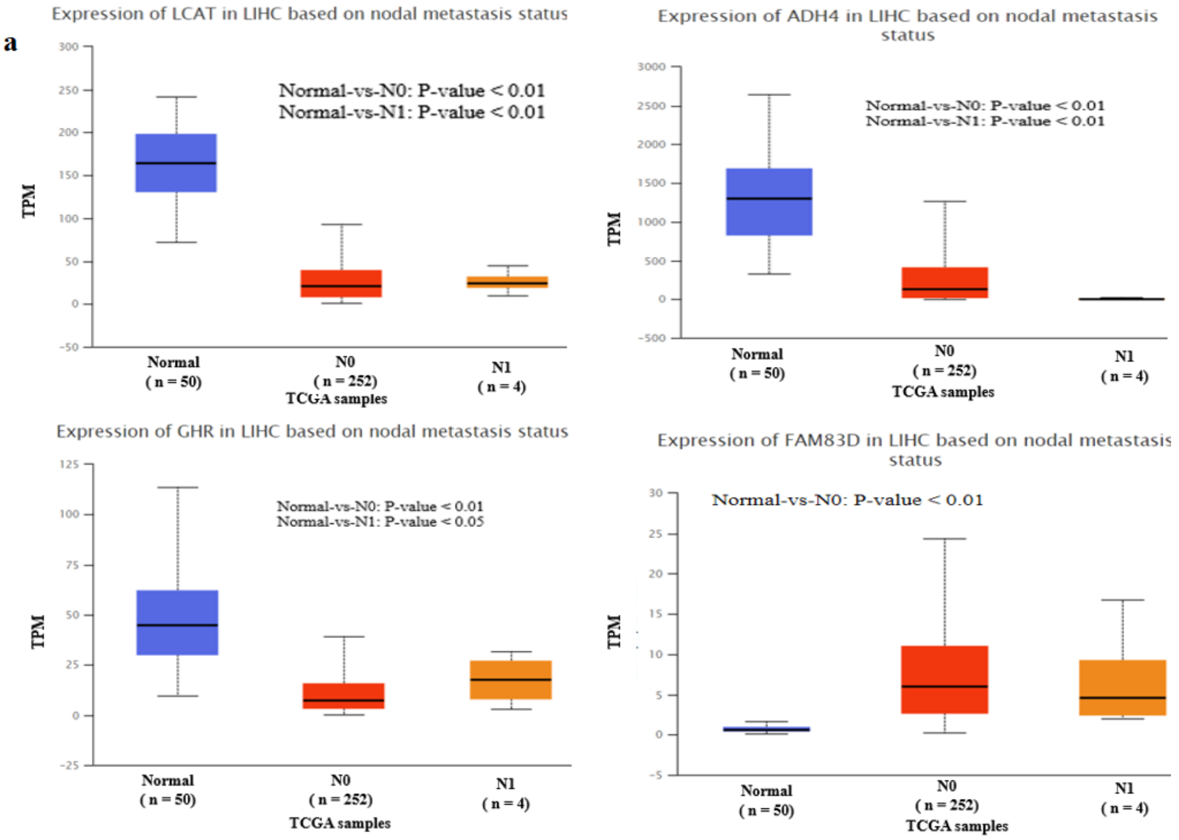

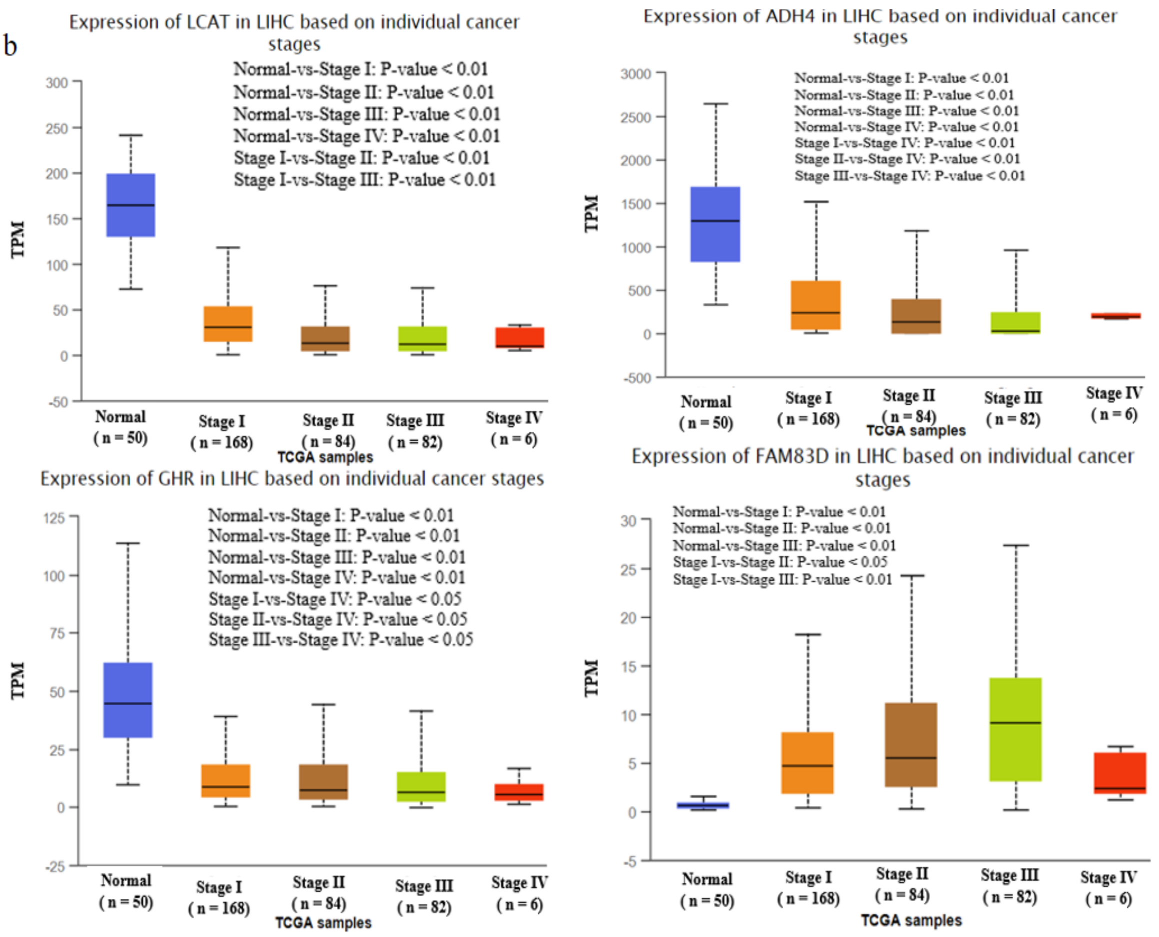
b
Expression of LCAT in LIHC based on individual cancer stages
Normal-vs-Stage I: P-value < 0.01
Normal-vs-Stage II: P-value < 0.01
Normal-vs-Stage III: P-value < 0.01
Normal-vs-Stage IV: P-value < 0.01
Stage I-vs-Stage II: P-value < 0.01
Stage I-vs-Stage III: P-value < 0.01
TPM
Normal (n = 50)
Stage I (n = 168)
Stage II (n = 84)
Stage III (n = 82)
Stage IV (n = 6)
TCGA samples
Expression of ADH4 in LIHC based on individual cancer stages
Normal-vs-Stage I: P-value < 0.01
Normal-vs-Stage II: P-value < 0.01
Normal-vs-Stage III: P-value < 0.01
Normal-vs-Stage IV: P-value < 0.01
Stage I-vs-Stage IV: P-value < 0.01
Stage II-vs-Stage IV: P-value < 0.01
Stage III-vs-Stage IV: P-value < 0.01
Expression of GHR in LIHC based on individual cancer stages
Normal-vs-Stage I: P-value < 0.01
Normal-vs-Stage II: P-value < 0.01
Normal-vs-Stage III: P-value < 0.01
Normal-vs-Stage IV: P-value < 0.01
Stage I-vs-Stage IV: P-value < 0.05
Stage II-vs-Stage IV: P-value < 0.05
Stage III-vs-Stage IV: P-value < 0.05
Expression of FAM83D in LIHC based on individual cancer stages
Normal-vs-Stage I: P-value < 0.01
Normal-vs-Stage II: P-value < 0.01
Normal-vs-Stage III: P-value < 0.01
Stage I-vs-Stage II: P-value < 0.05
Stage I-vs-Stage III: P-value < 0.01

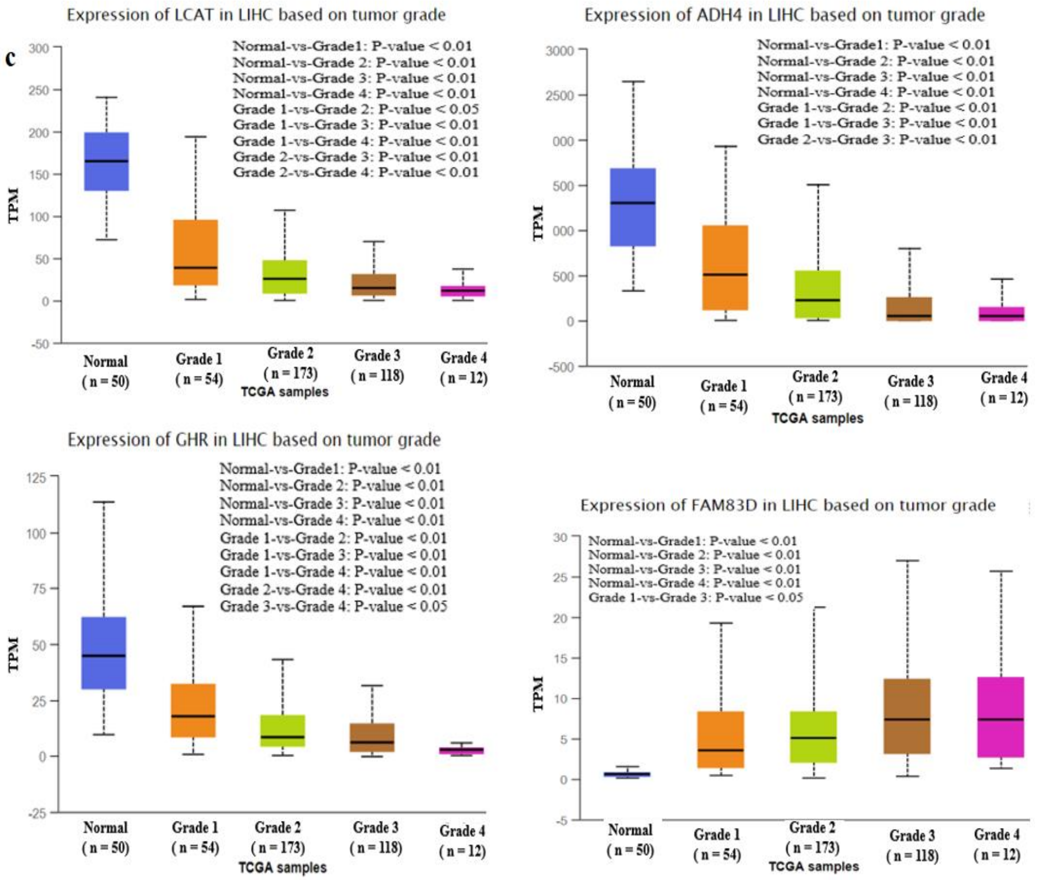
c
Expression of LCAT in LIHC based on tumor grade
Normal-vs-Grade1: P-value < 0.01
Normal-vs-Grade 2: P-value < 0.01
Normal-vs-Grade 3: P-value < 0.01
Normal-vs-Grade 4: P-value < 0.01
Grade 1-vs-Grade 2: P-value < 0.05
Grade 1-vs-Grade 3: P-value < 0.01
Grade 1-vs-Grade 4: P-value < 0.01
Grade 2-vs-Grade 3: P-value < 0.01
Grade 2-vs-Grade 4: P-value < 0.01
TPM
Normal (n = 50)
Grade 1 (n = 54)
Grade 2 (n = 173)
Grade 3 (n = 118)
Grade 4 (n = 12)
TCGA samples
Expression of ADH4 in LIHC based on tumor grade
Normal-vs-Grade1: P-value < 0.01
Normal-vs-Grade 2: P-value < 0.01
Normal-vs-Grade 3: P-value < 0.01
Normal-vs-Grade 4: P-value < 0.01
Grade 1-vs-Grade 2: P-value < 0.01
Grade 1-vs-Grade 3: P-value < 0.01
Grade 2-vs-Grade 3: P-value < 0.01
Expression of GHR in LIHC based on tumor grade
Normal-vs-Grade1: P-value < 0.01
Normal-vs-Grade 2: P-value < 0.01
Normal-vs-Grade 3: P-value < 0.01
Normal-vs-Grade 4: P-value < 0.01
Grade 1-vs-Grade 2: P-value < 0.01
Grade 1-vs-Grade 3: P-value < 0.01
Grade 1-vs-Grade 4: P-value < 0.01
Grade 2-vs-Grade 4: P-value < 0.01
Grade 3-vs-Grade 4: P-value < 0.05
Expression of FAM83D in LIHC based on tumor grade
Normal-vs-Grade1: P-value < 0.01
Normal-vs-Grade 2: P-value < 0.01
Normal-vs-Grade 3: P-value < 0.01
Normal-vs-Grade 4: P-value < 0.01
Grade 1-vs-Grade 3: P-value < 0.05

Supplementary Figure S1: The association between 4-gene signature expression level and various clinical characteristics. The association between gene expression level and clinical characteristics was identified by UALCAN bioinformatics tools on the basis of TCGA-HCC dataset (a-c). We obtained that lower expression of GHR, ADH4 and LCAT correlated with lower nodal metastasis (N0 and N1), stages (stage I, stage II, stage III, and stage IV) and tumor grades (grade1, grade 2, grade 3, and grade 4) relative to normal sample tissues. Meanwhile, higher expression of FAM83D closely related with higher nodal metastasis (N0 and N1), stages (stage I, stage II, stage III, and stage IV) and tumor grades (grade1, grade 2, grade 3, and grade 4) relative to normal tissue samples. The overall results indicated that expression level of the 4-gene signature associated with various cliniopathological stages in HCC patients. HCC, hepatocellular carcinoma.

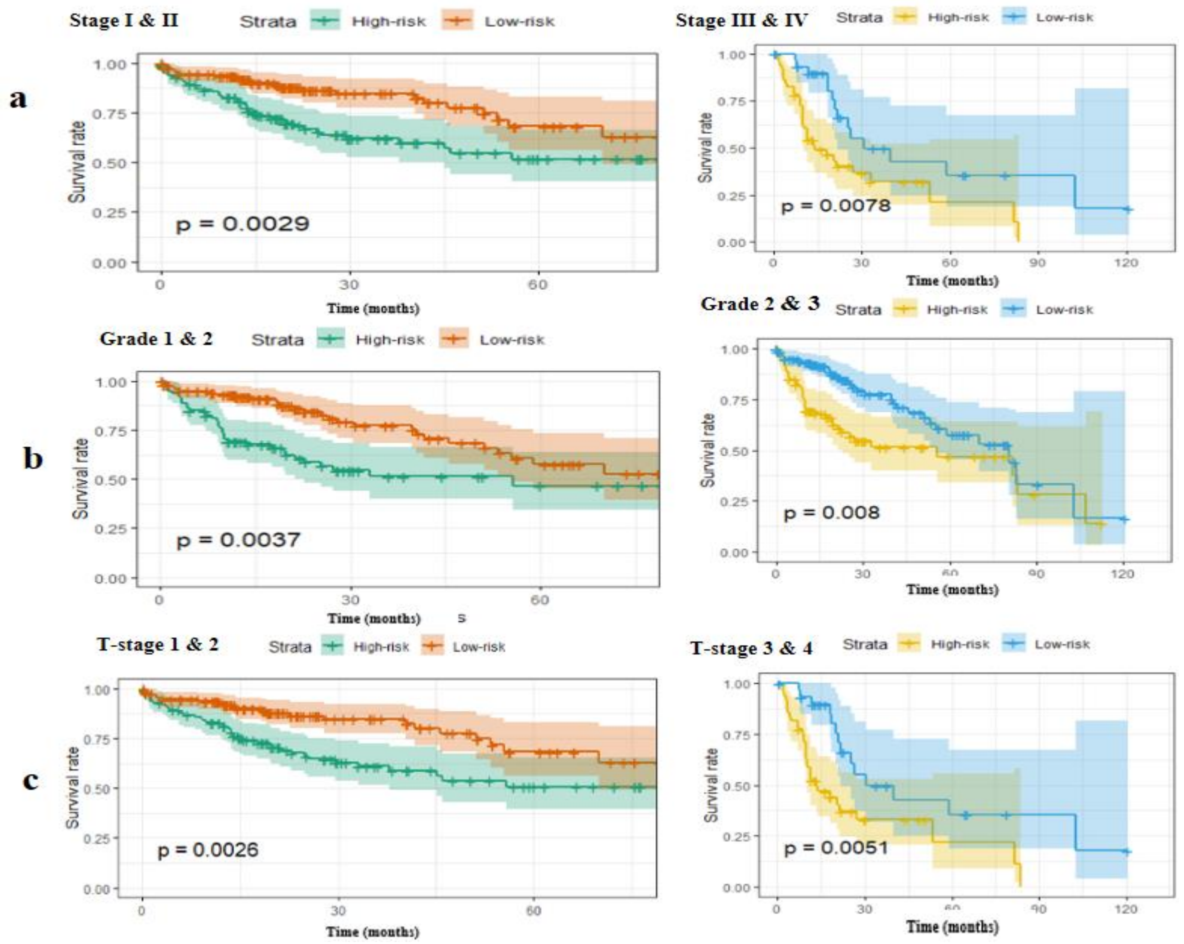

Supplementary Figure S2: Kaplan-Meier (KM) survival analysis of high-risk and low-risk stratified by different clinicopathological information. The association between risk expression level and clinical characteristics was analyzed by KM survival analysis the basis of TCGA-HCC dataset. We obtained that higher-risk correlated with lower survival rate in stage (I&II and III&IV) (a), grade (1&2 and 3&4) (b) and Tumor stage (1&2 and 3&4) (c). TCGA, The Cancer Genome Atlas; HCC, hepatocellular carcinoma.

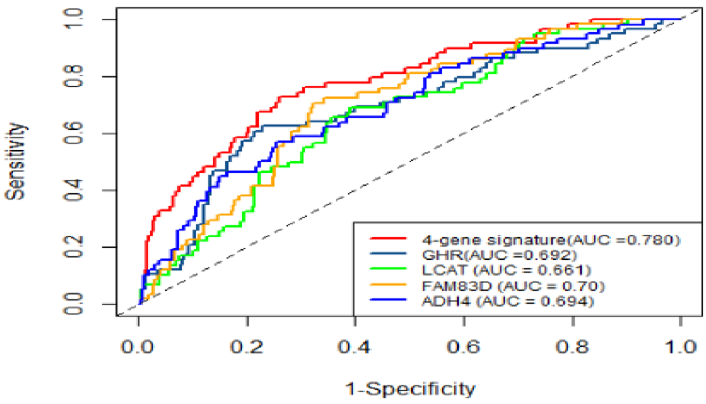

Supplementary Figure S3: Comparison the time dependent ROC analysis of 4-gene signature indicates that better risk stratification ability compared with individual gene signature. TCGA, The Cancer Genome Atlas; HCC, hepatocellular carcinoma.

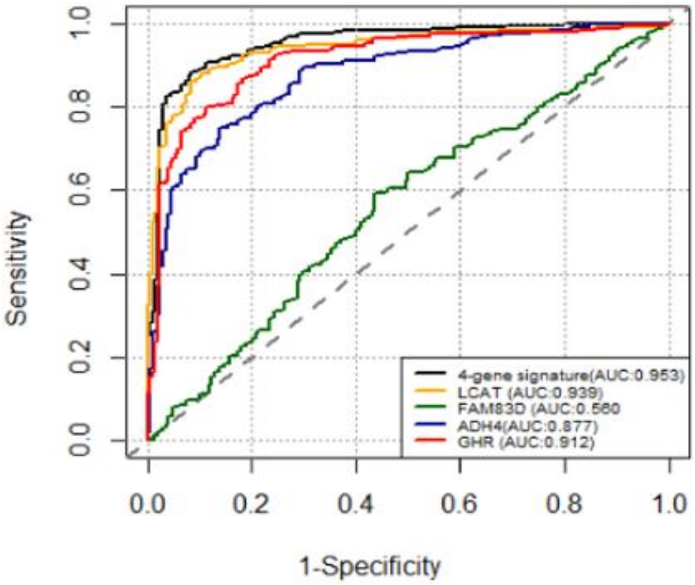

Supplementary Figure 4: The ROC curve for individual and combined gene signature diagnostic performance in classifying HCC and normal tissue samples based ICGC dataset. ICGC, International Cancer Genome Consortium; HCC, hepatocellular carcinoma.

Supplementary Table S1: Identification of consistently DEGs 238 downregulated and 101 upregulated genes across multiple Cohorts (TCGA-HCC, GSE112790, GSE84402 and GSE45267).

**DEGs**	**DEGs (|LFC| ≥ 1.5 and adjusted P-value = 0.01**
**Downregulated genes(N = 238)**	CLEC4M, CLEC4G, CLEC1B,CYP1A2, FCN2, MARCO, STAB2, MT1H, HAMP, PZP, CRHBP, CXCL14, MT1G, CNDP1, MT1M, FCN3, OLEC10, CYP3A4, C9, MT1F,

Copyright: © 2021 by the authors. Licensee MDPI, Basel, Switzerland. This article is an open access article distributed under the terms and conditions of the Creative Commons Attribution (CC BY) license (http://creativecommons.org/licenses/by/4.0/).

	SLCO1B3, TTC36, HHIP, CYP26A1, CD5L, KCNN2, MT1E, IGFALS, SLC22A1, SPO3, NAT2, ADRA1A, HAO2, THRSP, CYP2A7, HGFAC, GPM6A, MFSD2A, BCO2, CYP2A6, FOSB, APOF, ADH4, GLS2, CYP2E1, MT1X, CYP39A1, AVPR1A, C7, FOLH1B, CYP2C8, DCN, CFP, IL13RA2, OIT3, HEPN1, ESR1, GLYAT, HEPACAM, MOGAT2, CYP2B6, LPA, DNASE1L3, PLAC8, GYS2, LYVE1, PCK1, MT2A, SRD5A2, FAM134B, CLRN3, TMEM27, ANGPTL6, NNMT, ECM1, GBA3, CYP8B1, AADAT, FOS, FCGR2B, KBTBD11, MME, CXCL12, AKR1D1, SRPX, LIFR, FAM65C,CDH19,SLC10A1, BBOX1, LCAT, NPY1R, GREM2, HBA1, SPP2, RDH16, IDO2, F9, PDGFRA, ADH1C, HGF, KCND3, HPGD, CETP, TAT, CYP4A11, BHMT, SAA2, TDO2, CNTN3, SAA1, FXYD1, HSD11B1, CYP3A43, CYP4A22, CYP2C9, ADH1B, LY6E, BCHE, ANXA10SDS, GLYATL1, ZG16, GHR, IGF1, ACSM5, EGR1,C3P1,AFM, SAA4, CCL14, ACADLC6, MFAP3L, MBL2, GNMT, ABCA8, TFPI2, FBP1, ACSM3, PGL,YRP2, AGXT2, HBB, LECT2, STEAP4, ENO3, ASPA, HPD, FETUB, C8orf4, ALDOB, HRG, CFHR3, KMO, CPEB3, CYP4F2, EXPH5, ADH1A, ALDH8A1, PROZ, RND3, C8A, SLC27A5, TMEM45A, RCAN1, SOCS2, FAM13A, SERPINE1, PRG4, ALDH6A1, PLGLB2, AOX1, HPX, TRPM8APOA5, OGDHL, PLG, HBA2, SLC1A1, DNAJC12, CD1D, SLC13A5, CXCL2, GSTZ1, MAN1C1, SLC7A2, CFHR4, AZGP1, ATF5, PON1, MCC, MAT1A, ACSL1, FTCD, DMGDH, IL1RAP, CYR61, XDH, STEAP3, PBLD, KLKB1, ASS1, SLC4A4, MRC1, GRAMD1C, SLC38A4, C8B, C1R, CTH, CP, ITIH4, PDK4, SLCO1B1, RDH5, PON3, FOLH1, SLC1A2, SLC27A2, DHODH, EPHX2, HPR, CA2, ETFDH, CD14, FAM110C, ST3GAL6, ZGPAT, F11, SORL1, RCL1, CYP4V2, SRD5A1, DEPDC7, CDC37L1, MASP2, NAMPT, ACMSD, CYP4F3
**Upregulated genes(N = 101)**	PLXNC1, FEN1, ITGA2, RFC4, STXBP6, TOMM40L, MCM6, TYMS, UBE2S, FABP5, EPPK1, SQLE, PRR11, RBM24,TPX2, GMNN, CCNE2, ACSL4, CAP2, MAD2L1, KIF11, NUSAP1, LRRC1, FLVCR1, RAD51AP1, ROBO1, FAM83D, ECT2, CENPW, IGF2BP3, MCM2, S100P, DKK1, FAM72B, RACGAP1, MAGEA3, FAM72A, KIAA0101, HELLS, ZWINT, GINS1, EZH2, FANCI, SPINK1 AURKA, CENPK, DEPDC1B, OIP5, UBD, COCH, MAGEA6, KIF14, FAM72D, NQO1, SULT1C2, CTHRC1, CCNB1, GTSE1, RRM2, MKI67, PRC1, TRIP13, CCNA2, MDK, UHRF1, CDC6, DTL, CDCA3, CDK1, UBE2T, E2F8, HMMR, FOXM1, CENPA, PTTG1, BUB1B, DEPDC1, CENPF, PBK, NDC80, ANLN, ASPM, BUB1, DLGAP5, KIF4A, KIF2C, NEK2, UBE2C, KIF20A, NCAPG, CCNB2, COL15A1, TOP2A, TTK, MELK, DKN3,IQGAP3, BIRC5, CDC20,NUF2, GPC3

Supplementary Table S2. Results of significantly correlated genes with signature (GHR, ADH4, FAM83D and LCAT) based on TCGA-HCC.

**4- prognostic signature**	**Significantly correlated abnormal genes with four prognostic signature (Person correlation coefficient, r > 0.5 and P-value < 0.001)**
ADH4	ALDH8A1, SORD, ADHFE1, AASS, PAH, PIPOX, CRYL1, FMO3, HSD17B6, ALDH4A1, CAT, BDH1, HPD, ALDH6A1, BCKDHB, QDPR, CYB5A, CYP2E1, CDO1, ADI1, AOX1, ALDH2, DCXR, AADAC, ACSM2B, EHHADH, UGT2B10, ACSS3, SCP2, ACSL5, KNG1, F11, F12, PROZ, SERPINC1,F9, SERPING1, CPB2, PLG, PROC, ACOX2, ACOX1, GCDH, ACADSB, IVD, ACAD11, CADL, ABCB4, ECHDC2, ECHS1, ACAT1, SLC27A2, ACAA1, MBL2, MASP2, C6, C8A, CYP3A4, CYP2C9, HSD11B1, ADH1C, ADH1B, ADH1A, CYP1A2, TAT, NAT2, RDH16, MAT1A, ST3GAL6, CTH, BHMT, AKR1D1, SLC27A5, ACSL1, DMGDH, PCK1, AGXT2, CYP4F3, CYP4F2, CYP8B1, ACSM5
FAM83D	AURKA,SPC24,CCNE2,SPC25,NCAPH,CDCA2,SKA3,SKA1,CCNA2,CDCA5,ASPM, ERCC6L, CDCA3, KIF14, CDK1, CDC6, DSN1, CCNF, TPX2, CDC25C,CDC25A, CCNB1, CCNB2, ZWINT, KIF23, CKAP2, PLK1, NUSAP1,ANLN, STMN1, RACGAP1, KIF20A, NUF2, STIL,WDR62, GPSM2,NDC80,KIF2C, KIFC1, KIF4A, KIF11, KIF15, KIF18A, KIF20B, KIF18B, CENPE, CDC45 GINS4, MCM2, MCM10, MCM4, MCM6, TTK, CDC20, MAD2L1, BUB1, BUB1B, RRM2, TYMS
GHR	XDH,ALDH8A1,HSD17B6,CAT,ALDH6A1,CBR4,DBT,MUT,EPHX2, SCP2,KNG1,F11, KLKB1,ACADM,IVD,ET-FDH,ACAA2,ACAT1,C6,C8B,CYP2A6,TAT,CYP2C8,FBP1,HAO2, ALDOB, ACSL1, GBA3,CYP4F3, CYP4F2
LCAT	ALDH2, ECHDC2

Supplementary Table S2. The results of enrich GO terms of prognostic genes and their co-expressed DEGs.

**BP:Go terms**	**Count**	**P-value**	**FDR**	**Enriched genes**
GO:0051301~cell division	25	6.01E-13	9.74E-10	AURKA, SPC24, CCNE2, SPC25, NCAPH, CDCA2, SKA3, NSMCE2, SKA1, CCNA2, CDCA5, ASPM, ERCC6L, CDCA3, KIF14, CDK1, CDC6, DSN1,CCNF, TPX2, CDC25C, CDC25A, CCNB1, CCNB2, ZWINT
GO:0033539~fatty acid beta-oxidation using acyl-CoA dehydrogenase	9	3.40E-09	5.50E-06	ACOX2, ACOX1, GCDH, ACADSB, ACADM, IVD, ETFDH, ACAD11, ACADL
GO:0007067~mitotic nuclear division	17	9.63E-09	1.56E-05	CDK1, CCNF, NUF2, CDC25C, CDC25A, SPC24, SPC25, CCNB2, PLK1, ZWINT, CDCA2, NSMCE2, SKA3,SKA1, CCNA2, ASPM, ERCC6L
GO:0000281~mitotic cytokinesis	9	8.78E-08	1.42E-04	KIF23, CKAP2, PLK1, NUSAP1, ANLN, CEP55, STMN1, RACGAP1, KIF20A
GO:0007018~microtubule-based movement	12	1.16E-07	1.88E-04	KIF23, KIF14, KIF2C, KIFC1, KIF4A, KIF11, KIF15, KIF18A,KIF20B, KIF18B, CENPE, KIF20A
GO:0055088~lipid homeostasis	9	4.58E-07	7.43E-04	ACOX2, ACOX1, GCDH, ACADSB, ACADM, IVD, ACAD11, ACADL, ABCB4
GO:0007596~blood coagulation	11	5.36E-07	8.69E-04	KNG1, F11, F12, KLKB1, PROZ, SERPINC1, F9, SERPING1, CPB2, PLG, PROC

GO:0006270~DNA replication initiation	8	5.81E-07	9.41E-04	CCNE2, CDC6, CDC45, GINS4, MCM2, MCM10, MCM4, MCM6
GO:0006635~fatty acid beta-oxidation	9	1.05E-06	0.002	ACOX2, ACAA2, ACADM, EHHADH, ECHDC2, ECHS1, ACAT1, SLC27A2, ACAA1
GO:0007052~mitotic spindle organization	8	1.17E-06	0.002	CCNB1, SPC25, STIL, WDR62, GPSM2, AURKA, NDC80, STMN1
GO:0055114~oxidation-reduction process	25	2.08E-06	0.003	XDH, ALDH8A1, SORD, ADHFE1, AASS, PAH, PIPOX, CRYL1, FMO3, HSD17B6, ALDH4A1, CAT, BDH1, HPD, ALDH6A1, BCKDHB, QDPR, CBR4, CYB5A, CYP2E1, CDO1, ADI1,AOX1, ALDH2, DCXR
GO:0008152~metabolic process	12	2.24E-05	0.036	AADAC, ACSM2B, DBT, MUT, EHHADH, BCKDHB, EPHX2, TKT, UGT2B10, ACSS3, SCP2, ACSL5

Supplementary Table S3: Description of genomic datasets used in our study.

Dataset	Source	Sample size (HCC)	Sample size (normal/control)	Application
GSE112790	GEO	N = 183	N = 15	DEGs Identification
GSE84402	GEO	N = 14	N = 14	DEGs Identification
GSE45267	GEO	N = 48	N = 39	DEGs Identification
HCC	TCGA	N = 371	N = 50	DEGs, prognostic and diagnostic model Identification
LIRI-JP	ICGC	N = 243	N = 202	DEGs, prognostic and diagnostic model validation
CMUH-dataset	CMUH	N = 168	N = 12	DEGs, prognostic and Diagnostic model verification

Abbreviations: GEO, Gene Expression Omnibus; TCGA, The Cancer Genome Atlas; ICGC, International Cancer Genome Consortium; CMUH, China Medical University Hospital.
